# Site-Specific Distribution of CD68-Positive Microglial Cells in the Brains of Human Midterm Fetuses: A Topographical Relationship with Growing Axons

**DOI:** 10.1155/2013/762303

**Published:** 2013-12-29

**Authors:** Kwang Ho Cho, Jin Sung Cheong, Ji Hyun Kim, Hiroshi Abe, Gen Murakami, Baik Hwan Cho

**Affiliations:** ^1^Department of Neurology, Jeonbuk Regional Cardiocerebrovascular Disease Center, Institute of Wonkwang Medical Science, Wonkwang University School of Medicine, Iksan 570-711, Republic of Korea; ^2^Department of Anatomy, Chonbuk National University School of Medicine, 634-18 Geumam-dong, Deokjin-gu, Jeonju 561-712, Republic of Korea; ^3^Department of Anatomy, Akita University School of Medicine, Akita 010-8502, Japan; ^4^Division of Internal Medicine, Iwamizawa Kojin-kai Hospital, Iwamizawa 068-0833, Japan; ^5^Department of Surgery, Chonbuk National University School of Medicine, 634-18 Geumam-dong, Deokjin-gu, Jeonju 561-712, Republic of Korea

## Abstract

Using 5 fetuses of gestational age (GA) of 15-16 weeks and 4 of GA of 22–25 weeks, we examined site- and stage-dependent differences in CD68-positive microglial cell distribution in human fetal brains. CD68 positive cells were evident in the floor of the fourth ventricle and the pons and olive at 15-16 weeks, accumulating in and around the hippocampus at 22–25 weeks. At both stages, the accumulation of these cells was evident around the optic tract and the anterior limb of the internal capsule. When we compared CD68-positive cell distribution with the topographical anatomy of GAP43-positive developing axons, we found that positive axons were usually unaccompanied by CD68-positive cells, except in the transpontine corticofugal tract and the anterior limb of the internal capsule. Likewise, microglial cell distribution did not correspond with habenulointerpeduncular tract. Therefore, the distribution of CD68-positive cells during normal brain development may not reflect a supportive role of these microglia in axonogenesis of midterm human fetuses.

## 1. Introduction

Microglia are the parenchymal mononuclear phagocytes of the central nervous system (CNS) and progressively populate the CNS during the fetal period of development in man, most conspicuously during the second trimester of life. During development, microglia appear in developing fiber tracts throughout the white matter before myelinogenesis in vivo [1]. Although microglia use white matter tracts as migratory pathways to the cerebral cortex [2], the main phase of migration does not occur until the second trimester in the telencephalon [3]. Although microglial hot spots are densely distributed in the subplate at 10–12 weeks, microglial cells at 19–24 weeks are restricted to (1) the ditelencephalon fissure, (2) being around the thalamus, (3) the corona radiata around the developing putamen, (4) the midline septal area, and (5) the optic tract [4, 5]. These findings suggest that microglial cells phagocytose specific transient axons such as parts of thalamocortical projections. Indeed, microglia were reported to eliminate exuberant transcallosal projections during the development of the cat brain [6]. However, because of stages much earlier than myelination, myelin phagocytosis [7] is unlikely to occur in midterm fetuses.

The cerebral white matter during the last half of human gestation expresses high levels of GAP-43, indicating active outgrowth of axons during this mid- to late gestation stage [8]. Therefore, it is possible that a transient elevation of activated microglia during the time frame of active axonal outgrowth may reflect either a supportive role of these microglia in axonogenesis or a role in “pruning” overabundant axons by activated microglia. We used two immunological markers to assess the density of activated microglia, namely, CD 68 and major histocompatibility complex-class II (MHC-II). CD68-positive microglial cells have frequently been observed in human fetal white matter [9–11].

We hypothesized that, between 12 and 18 weeks, sites of elimination of nerve fibers by microglial cells move from the subplate of the telencephalon to the diencephalon and more caudal areas. Müller and O'Rahilly [12] described key tracts of the embryonic human brain from stage 8 to stage 23, such as the habenulointerpeduncular, preoptic-hypothalamotegmental, accessory optic, and mammillotegmental tracts. These tracts are characterized by the early development but become less evident in the later stages. The habenulointerpeduncular tract or fasciculus retroflexus provides major monoaminergic input to the midbrain and is well developed in adult fish and rats [13]. Although these tracts were described in sagittal sections [12], excellent atlases by Bayer and Altman [14] showed that the habenulointerpeduncular tract can be identified also in frontal sections, starting from the medial aspect of the thalamus and running inferiorly and caudally between the red nucleus and the oculomotor complex. These well-developed tracts at these stages, in and around the diencephalon, are likely to be targets of elimination by microglial cells. Consequently, this study was designed (1) to compare the distribution of CD68-positive microglia in fetuses of gestational ages (GA) of 15-16 and 22–25 weeks and (2) to interpret the areas of accumulation relative to the topographical anatomy of GAP43-positive developing axons [15, 16].

## 2. Materials and Methods

The study was performed in accordance with the provisions of the Declaration of Helsinki 1995 (as revised in Edinburgh 2000). We assessed the histology of nine paraffin-embedded fetuses: 5 at an estimated GA of 15-16 weeks (102–120 mm crown-rump length (CRL)) and 4 at an estimated GA of 22–25 weeks (180–200 mm CRL). With the agreement of the families concerned, these specimens had been donated to the Department of Anatomy, Chonbuk National University in Korea, and their use for research was approved by the university ethics committee. The fetuses had been obtained by induced abortions. After abortion, each of the mothers was personally informed by an obstetrician about the possibility of donating the fetus for research; no attempt was made to encourage donation. Because the samples were collected without personal identifiers, it was not possible to trace any of the families concerned.

The donated fetuses were fixed in 10% w/w neutral formalin solution for more than 1 month (3 months at maximum). After division into the head and neck, the thorax, the abdomen, the pelvis, and the four extremities, all parts were decalcified by incubating at 4°C in 0.5 mol/L EDTA (pH 7.5; decalcifying solution B; Wako, Tokyo) for 1–3 days, depending on the size of the body part. At 50 or 100 micrometer intervals, depending on size, specimens of the head were processed for sagittal or horizontal sections, each 5 micrometers thick. These sections included not only the brain but also any surrounding structures, including the eye, the ear, and the nose.

Most sections were stained with hematoxylin and eosin (HE), whereas others were used for immunohistochemical assays. The primary antibodies used were (1) mouse monoclonal anti-human CD68 KP1 (diluted 1 : 100, Dako M0814, Glostrup, Denmark); (2) mouse monoclonal anti-human HLA-class II DR alpha chain (1 : 100, Dako M0746); (3) mouse monoclonal anti-human vimentin (1 : 10; Dako M7020); (4) mouse monoclonal anti-human glial fibrillary acidic protein of GFAP (1 : 100; Nichirei 422261, Tokyo, Japan); (5) mouse monoclonal anti-human growth associated protein-43 (GAP43; 1 : 8000; Sigma-Aldrich, St. Louis, USA); (6) mouse monoclonal anti-human proliferative cell nuclear antigen (PCNA; dilution 1 : 1000; Abcom, Cambridge, UK). The samples were not autoclaved prior to treatment because of the loose nature of the fetal tissues. Secondary antibody (Dako Chem Mate Envison Kit) was labeled with horseradish peroxidase (HRP), and antigen-antibody reactions were detected via the HRP-catalyzed reaction with diaminobenzidine. All samples were counterstained with hematoxylin. We tried to perform a pair of different immunohistochemistry (such as CD68 and GFAP) using adjacent sections, but we often used near (not adjacent) sections due to a failure of the sectioning and/or immunostaining.

## 3. Results

**Figure 1:**
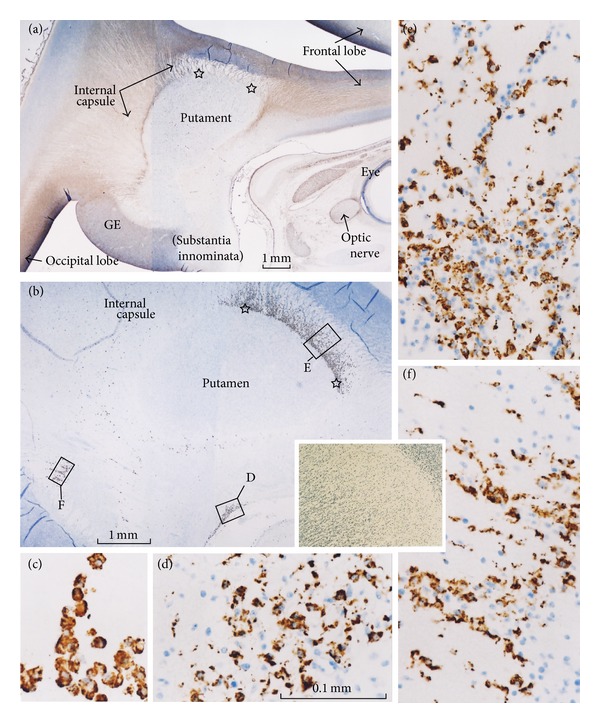
Distribution of CD68-positive microglial cells in and around the internal capsule at 15 weeks. Panel (a) immunohistochemistry of vimentin, and panel (b) immunohistochemistry of CD68 display near sections. Microglial cell accumulation in the corona radiata is clearly seen in the anterosuperior aspect of the putamen (stars in panel (b)). Insert at the bottom of panel (b) is a negative control showing the corona radiata. Panel (c) exhibits CD68-posiitve cells in the choroid plexus. Panels (d), (e), and (f), corresponding to squares in panel (b), display higher magnification views of CD68-positive microglial cells.

**Figure 2:**
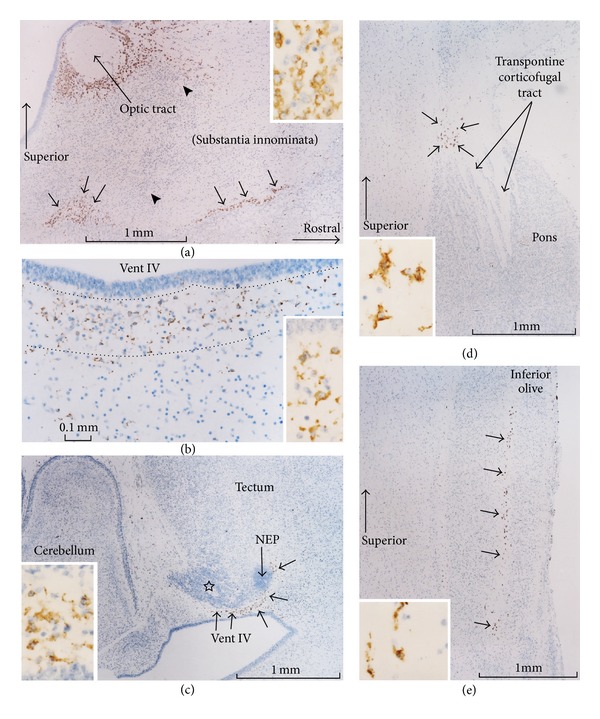
Distribution of CD68-positive microglial cells in a brain at 16 weeks. Immunohistochemistry of CD68. Panel (a) displays a cortex around the optic tract. CD68-positve cells are distributed along the optic tract and around the substantia innominata (arrows). Arrowheads in Panel (a) indicate vessels containing no cells. Panel (b) exhibits a laminar distribution (sandwiched by dotted lines) of CD68-positive cells at the floor of the fourth ventricle (vent IV). Panel (c) displays the tectum and cerebellum: CD68-positive cells are distributed around a cluster of developing neurons (star) from the neuroepithelium (NEP) facing the fourth ventricle (vent IV). Panel (d) exhibits a cluster of CD68-positve cells at the superior end of the pons. Panel (e) displays a linear distribution of CD68-positve cells in the inferior olive. Insert in each panel shows the higher magnification of microglial cells.

**Figure 3:**
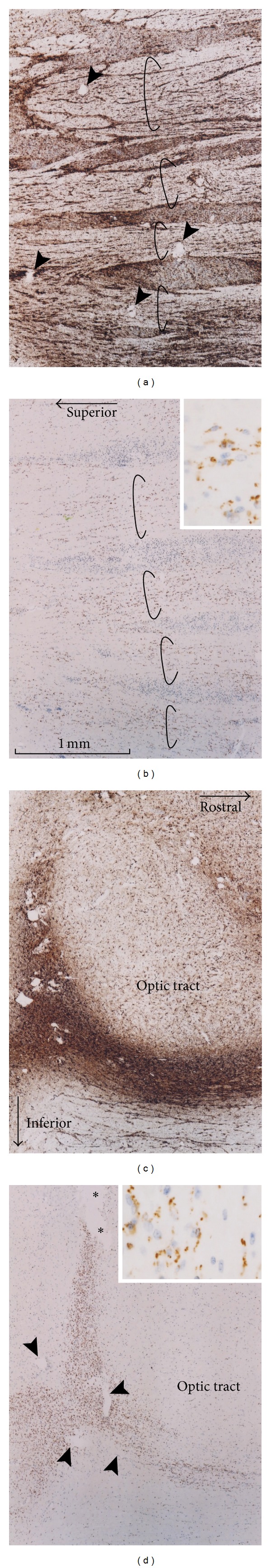
Distribution of CD68-positive microglial cells in a brain at 25 weeks. Panels (a) and (c) are immunohistochemistry of glial fibrillary acidic protein, while panels (b) and (d) are that of CD68. Panels (a) and (b) (near sections) display the corona radiata in the anterosuperior side of the putamen: CD68-positive cells are seen along nerve fiber bundles (bundled by semicircles). Panels (c) and (d) (near sections) exhibit the optic tract: CD68-positive cells are accumulated in the posteroinferior side of the tract. Arrowheads in panels (a) and (d) indicate vessels containing no cells. Insert in panels (b) and (d) shows the higher magnification of microglial cells.

**Figure 4:**
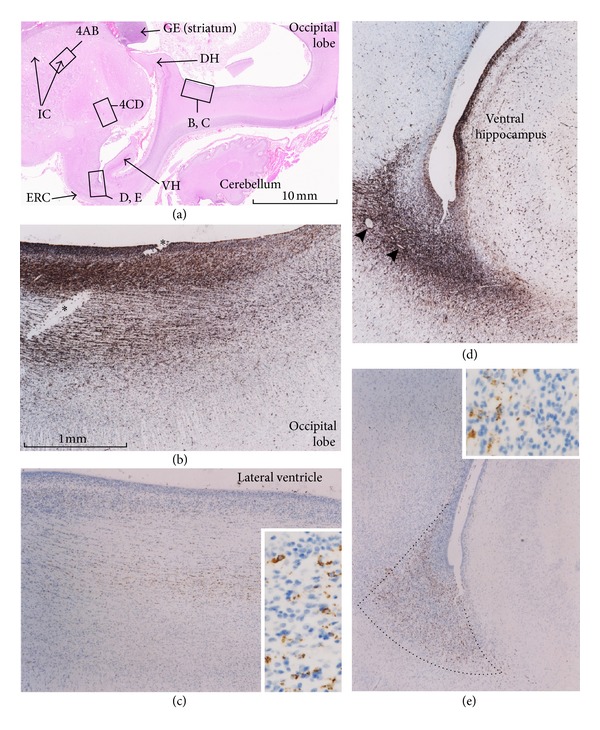
Distribution of CD68-positive microglial cells in a brain at 22 weeks. Panel (a) (HE staining) includes the ventral hippocampus (VH) and the entorhinal cortex (ERC) at the anterior part as well as the occipital lobe and the dorsal hippocampus (DH) at the posterior part. Panels (b)–(e) correspond to squares in panel (a). Panels (b) and (d) are immunohistochemistry of glial fibrillary acidic protein, while panels (c) and (e) are that of CD68. Panels (b) and (c) (near sections) display a laminar distribution of CD68-positve cells in the occipital lobe cortex. Asterisks in panel (b) indicate a damage of section during histological procedure. Panels (d) and (e) (near sections) exhibit a cluster of CD68-positve cells facing a recess of the lateral ventricle along the ventral hippocampus. Insert in panels (c) and (e) shows the higher magnification of microglial cells. GE, germinal eminence; IC, internal capsule.

**Figure 5:**
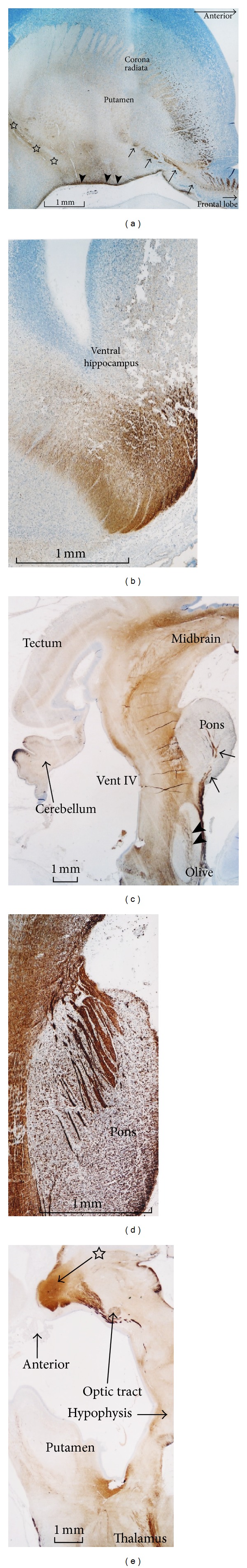
GAP43-positive nerve fibers in a brain at 16 weeks. The same specimen as shown in Figure 2. Panel (a) displays GAP43-positive nerve fibers in and around the putamen: arrows indicate a bundle from or to the frontal lobe cortex; stars indicate a candidate of the habenulointerpeduncular tract; arrowheads indicate a positive zone along the pia. The anteroinferior part of the corona radiata also contained GAP43-positive fibers (see also Figures 1(a) and 1(b)). Panel (b) exhibits a cluster of GAP43-positve fibers near the ventral hippocampus (see also Figures 4(d) and 4(e)). Panel (c) includes the cerebellum, tectum, midbrain, pons, and inferior olive: arrows indicate the transpontine corticofugal tract; arrowheads indicate fibers passing through the olive (see also Figure 2(e)). Panel (d) shows the rostral part of the transpontine corticofugal tract (see also Figure 2(d)). Panel (e) displays the optic tract and a candidate of the rostral end of the fornix (star; see also Figure 2(a)).

**Figure 6:**
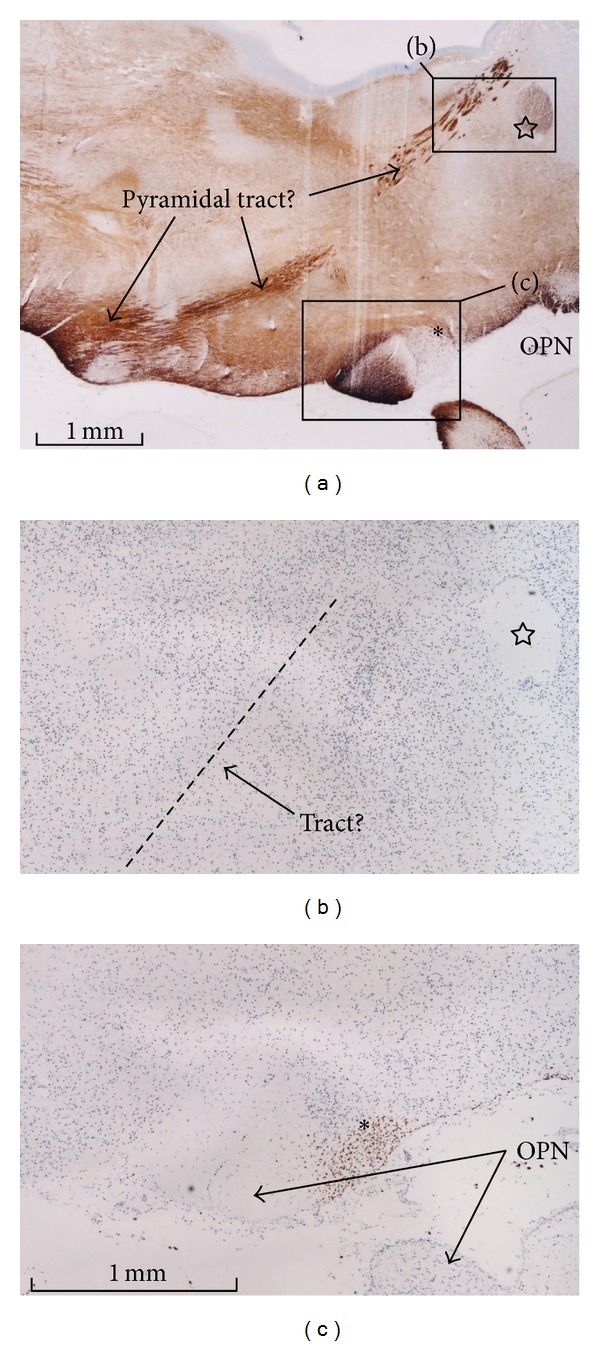
A GAP43-positive developing fiber tract does not contain CD68-positve cells. A brain at 15 weeks. Panel (a) (GAP43 immunohistochemistry) includes the optic nerve (OPN) and an early form of the pyramidal tract (center of the panel, obliquely running). Panels (b) and (c) correspond to higher magnification view of squares in panel (a). Star in panels (a) and (b) indicates a candidate of the anterior commissure. The pyramidal tract expresses GAP43 strongly (panel (a)), but it does not contain CD68-positve cells (panel (b)). In contrast, an area adjacent to the optic tract (asterisk in panels (a) and (c)) contained a cluster of CD68-positve cells (panel (c)).

**Figure 7:**
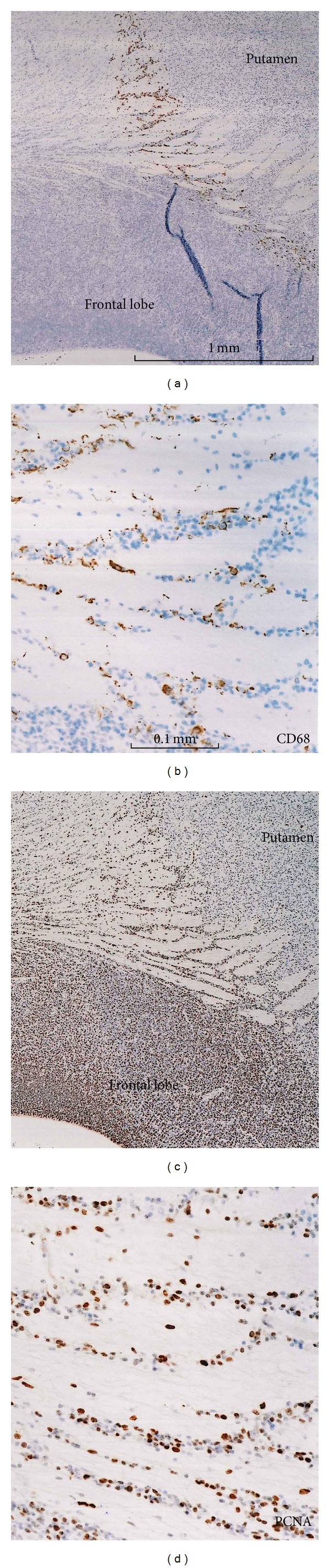
Immunohistochermistry for CD68 and PCNA. Panel (a) (CD68) and panel (c) (PCNA) display near sections (15 weeks) including the anterior end of the putamen. Panels (b) and (d) are higher magnification view of the central parts of panels (a) and (c), respectively. The CD68-positive cells (panel (a)) appear to overlap with a reticular configuration of PCNA positive cells. Note the frontal cortex expressing PCNA strongly (panel (c)). Panels (a) and (c) (or (b) and (d)) were prepared at the same magnification (scale bar in panels (a) and (b)).

At 15-16 weeks, CD68-positive microglial cells had accumulated (1) in parts of the internal capsule, including the corona radiata in the anterosuperior and posteroinferiorsides of the putamen, that is, the anterior and posterior limbs of the internal capsule (Figure 1); (2) around the optic tract or in the substantia innominata (Figure 2(a)); (3) at several sites beneath the neuroepithelium of the floor of the fourth ventricle (Figure 2(b)); (4) on the inferior side of the tectum near the fourth ventricle (Figure 2(c)); (5) at the superior end of the pons (Figure 2(d)); (6) in the inferior olive (Figure 2(e)); (7) beneath the neuroepithelium of the septum area along the cavum septi; (8) in the posterior commissure and around the habenula. Each of these sections, including the floor of the fourth ventricle, demonstrated 2–5 clusters of CD68-positive cells beneath the neuroepithelium, with the positive cells forming a pair of longitudinal belts along the left and right aspects of the mid-sagittal notch of the brain stem. The anterior commissure as well as the roots of the cranial nerves contained no or few positive cells. The cerebellum, including the eosinophilic lamina dissecans, did not contain any positive cells, but the developing cerebellar peduncles near the pons contained a cluster of positive cells. The microglial cells in the brain were round, fiber-like, or amoeboid in shape (Figures 1 and 2), whereas the pia and choroid plexus contained large, round cells expressing CD68 (Figure 1(c)).

Vimentin- or GFAP-positive fiber bundles of the coronal radiata were much thicker at 22–25 weeks than at 15-16 weeks (Figure 3(a) versus Figure 1(a)). Sites of accumulation of CD68-positve cells were similar in the two sets of fetuses, with fetuses of GA 22–25 weeks containing cells (1) in the corona radiata of the anterior limb of the internal capsule (Figure 3(b)) and (2) around the optic tract or in the substantia innominata (Figure 3(d)). However, no positive cells were observed in the posterior limb of the internal capsule, along the fourth ventricle, or at and around the pons and olive. Instead, at 22–25 weeks, CD68-positive microglial cells appeared in the occipital lobe cortex near the dorsal hippocampus (Figure 4(c)) as well as in the endorhinal cortex at a bottom of a recess of the lateral ventricle along the ventral hippocampus (Figure 4(e)). The CD68-positive cells in the occipital lobe and entorhinal cortex appeared to be arrayed along GFAP-positive fibers (Figures 4(b) and 4(d)). The positive cells at 22–25 weeks were smaller than those at 15-16 weeks, with the former having a relatively simple, dot-like shape.

All of the CD68-positive microglial cells, at both 15-16 and 22–25 weeks, including osteoclasts in the cranial base, were negative for HLA-class II DR. However, a few large, reticular cells in the occipital lymph nodes strongly expressed CD68. In contrast to Monier et al. [4], we did not find microvessels filled with CD68-positive cells (Figures 2(a), 3(a), 3(d), and 4(d)). Differences in CD68-positive cell distribution between stages are summarized in Table 1.

All of the 22–25-week samples were negative immunohistochemically for GAP43, partly due to the long preservation of these specimens in formalin solution. At 15-16 weeks, GAP43-positive fibers were evident in the subplate of the neocortex, including the frontal lobe (Figure 5(a)), the fornix (Figure 5(e)), the anterior and posterior commissures, and the cerebellum, especially its peduncles. However, positive fibers were not so abundant in the early form of the internal capsule or pyramidal tract (Figure 6). GAP43-positive fiber bundles were also observed in and along the putamen (Figure 5(a)) and thalamus, in the entorhinal cortex at the bottom of a recess of the lateral ventricle along the ventral hippocampus (Figure 5(b)), in the corticofugal fibers passing through and along the pons and olive (Figures 5(c) and 5(d)), and around the optic tract (Figure 5(e)). A candidate for the habenulointerpeduncular tract, crossing the diencephalon on the dorsal side of the putamen, was also positive for GAP43 (Figure 5(a)). However, with the exception of the corticofugal fibers passing through and along the pons and olive (Figures 2(d) and 2(e)), these GAP43 positive fibers did not contain any CD68-positive cells. Finally, immunohistochemistry of PCNA suggested that CD68-positive cells were in a proliferative state (Figure 7) although the analysis was conducted using semiserial sections (not serial).

## 4. Discussion

Although microglia are identified in the human CNS as early as embryonic development, the main phase of migration does not occur until the second trimester in the telencephalon [3], and it constitutively exhibits at least three types of morphology (i.e., amoeboid, ramified, and intermediate) [2]. The cerebral white matter during this period is potentially in a more “activated” state [11], because these cells may participate in developmental processes in the mid- to late-gestation organization of the brain, including vascularization [2], involution of the germinal matrix [17], programmed cell death [18], axonal development [8], and myelination [19]. The finding of a higher proportion of amoeboid microglia during the latter half of gestation suggests that the cerebral white matter during this period is potentially in a more “activated” state, a notion further supported by us with use of the antibody directed against CD68 to quantify activated microglial density. As with tissue injury, microglial upregulation of CD68 in response to programmed cell death during development prepares the cell to become phagocytic [20], whereas upregulation of MHC-II plays a role in immunity against foreign antigens in infection [21].

We observed differences in the distribution of CD68-positive cells between fetal stages. CD68-positive cells were evident in the floor of the fourth ventricle and the pons and olive at 15-16 weeks, whereas they accumulated in and around the hippocampus at 22–25 weeks. The accumulation of these cells around the optic tract may not correspond to well-developed tracts at these stages but to a part of the commissural plate [12]. Likewise, only a limited part of the habenulointerpeduncular tract seemed to contain CD68-positive cells. When we compared CD68-positive cell distribution with the topographical anatomy of GAP43-positive developing axons, we found that most of the GAP43-positive axons were negative for CD68 expression, with a few exceptions, such as the transpontine corticofugal tract and the anterior limb of the internal capsule. The corticofugal fibers passing through the pons and olive may correspond to the early form of the pyramidal tract [14]. In contrast to our working hypothesis, discrepancies between GAP43 and CD68 immunopositivity were observed in the subplate of the neocortex, the anterior commissure, and peduncles of the cerebellum, all of which expressed GAP43 strongly but contained no or few CD68-positive cells. Hyaluronic acid has been found to facilitate cell movement in the fetal brain by weakening cell attachment to adhesive substrates and by creating hydrated pathways for migrating cells [22]. We recently reported the distribution of hyaluronan in the human fetal brain [23]. Although CD68-positve cells were apparently absent from hyaluronan-rich areas, such as the subplate and putamen, hyaluronan-poor areas did not always correspond to the localization of CD68-positve cells.

Another striking difference between stages was the size and shape of CD68-positive microglial cells, with the large, round, highly proliferative cells present at 15-16 weeks (Figure 7) but absent at 22–25 weeks. However, in contrast to Wierzba-Bobrowicz et al. [24, 25] and Billiards et al. [11], all CD68-positive cells in the present study were consistently negative for HLA-DR. Thus the lack of MHC-II immunostaining of microglia in our cases reinforces classification of these cells as normative (i.e., not activated in response to infection and pathological inflammation).

We therefore question the basic concept that a specific pattern of distribution of CD68-postive cells corresponds to a functional state, such as the elimination of nerve fibers. The site- and stage-dependent distribution of CD68-positive cells may not reflect the supportive role of these microglia in axonogenesis in midterm human fetuses but may have a more specific role in histogenesis and modelling of the CNS during the development.

## Figures and Tables

**Table 1:** Differences in CD68-positive cell distribution between stages.

	15-16 weeks	22–25 weeks
Occipital lobe to the dorsal hippocampus	−	+
Corona radiata, anterior limb	+ +	+
Corona radiata, posterior limb	+	−
Around the optic tract (substantia innominata)	+	+
Ventral hippocampus	−	+
Habenulointerpeduncular tract	−	−
Floor of the fourth ventricle	+	−
Pons and olive	+	−
